# Effect of concurrent use of anti-retroviral therapy and levonorgestrel sub-dermal implant for contraception on CD4 counts: a prospective cohort study in Kenya

**DOI:** 10.7448/IAS.16.1.18448

**Published:** 2013-03-01

**Authors:** David Hubacher, Jennifer Liku, James Kiarie, Joel Rakwar, Peter Muiruri, Jackline Omwenga, Pai-Lien Chen

**Affiliations:** 1 FHI 360, Durham, NC, USA; 2 FHI 360, Nairobi, Kenya; 3 Institute of Tropical and Infectious Diseases, University of Nairobi, Nairobi, Kenya; 4 Kenyatta National Hospital, Nairobi, Kenya

**Keywords:** ART, levonorgestrel sub-dermal implant, contraception, disease progression, interactions, CD4, prospective cohort study, effectiveness

## Abstract

**Introduction:**

Simultaneous use of contraceptive hormones and anti-retroviral therapy (ART) may theoretically lessen the effectiveness of both. Women on ART need assurance that hormonal contraception is safe and effective. The sub-dermal implant is an ideal product to study: low and steady progestin release and no adherence uncertainties. We sought to determine if the medications’ effectiveness is compromised.

**Methods:**

We conducted a prospective cohort study among women on first line ART (stavudine or zidovudine and lamivudine+nevirapine). We recruited new implant users and matched them to women not using hormonal contraception, based on age and baseline CD4. Participants were followed prospectively for up to two years, recording serial CD4 measures and medical histories. We used generalized growth curve models and Wald chi-square tests to compare changes in CD4 counts across study groups. Prospective CD4 measures were censored (excluded) if any of the following events occurred: change in ART, implant removal or use of any hormonal contraception among controls. We examined incidence of opportunistic infection and pregnancy.

**Results:**

We matched 48 implant users to 33 non-hormonal controls. Over time, CD4 counts for both groups rose slightly but did not deviate significantly from each other (*p*=0.44). Opportunistic infection rates did not differ between the groups. None of the implant users and one of the non-hormonal controls became pregnant during follow-up.

**Conclusions:**

This small study found concurrent use of contraceptive implants and ART to be safe and effective. Although other hormonal contraceptive products and ART regimens may interact in unknown ways, the results of this study are reassuring.

## Introduction

Women living with HIV using combination anti-retroviral therapy (ART) need safe and effective contraceptive options to avoid unintended pregnancy. Hormonal contraceptives, including orals and injectables (such as depot medroxyprogesterone acetate, DMPA), are the most widely used type of modern method in sub-Saharan Africa (approximately 15 million current users in the region) [1]. ART is used by approximately three million women there [2].

One concern about hormonal contraception is that it may accelerate HIV-related disease progression by interfering with the body's natural immune responses. Seminal research examining viral set points, changes in CD4 counts and mortality has raised concerns [3–5], but systematic reviews find no strong associations between hormonal contraception and disease progression [6–8]. A second concern involves concomitant use of ART and hormonal contraception, and the potential for interactions and compromised effectiveness of each [9].

Valid measurement of correct and consistent use of hormonal contraception is difficult for products that require on-going user adherence; thus, contraceptive method failure (pregnancy) in the presence of ART use can be misinterpreted. But sub-dermal implants are inserted and removed by clinicians; this circumvents adherence problems and provides more certainty about actual use. We conducted a prospective cohort study in Kenya to examine how concurrent use of hormonal contraceptive implants and ART might lessen the effectiveness of both medications.

## Methods

The study was reviewed and approved by two Institutional Review Boards at FHI 360 in North Carolina, USA, and the Kenya Medical Research Institute, Nairobi, Kenya. Women voluntarily enrolled through a written informed consent process. All participants were receiving standard HIV-related care at the comprehensive care centre of Kenyatta National Hospital.

We recruited 60 women on ART who wanted to use a levonorgestrel (LNG) contraceptive implant (the product is marketed as Jadelle^®^). The following additional inclusion criteria were applied: aged between 18 and 44 years, last CD4 count of at least 200 cells, at least six months on a first line therapy of stavudine or zidovudine plus lamivudine+nevirapine, sexually active and willing to continue ART. (We chose first line therapies to maximize participation, though other ART regimens could be studied, since contraindications to implant use are non-existent.). We applied the following exclusion criteria to allow enough of a wash-out period for previous hormonal contraception: ≤ five months since last DMPA injection, ≤ two months since last dose of oral contraceptives, ≤ two months since removal of an LNG intrauterine device and ≤ two months since removal of contraceptive implant. We excluded women who were not appropriate candidates for an implant: currently pregnant, desire for pregnancy in the next 12 months, surgically sterilized (including partner vasectomy) and medical contraindications for implant use. To avoid unstable health histories that might require other interventions, we excluded women who were pregnant within the past six months, women currently taking rifampicin and women in unstable WHO stage 3 or 4 HIV disease. On the day of enrolment, a new blood draw was done and sent to the lab for analysis and the sub-dermal implant was inserted. Once the new CD4 count was available and confirmed to be at least 200 cells, we added the implant user to a list of women needing a matched control and recorded participant ID number, current age and CD4 count.

We recruited a cohort of matched controls who were not using hormonal contraception. We applied three matching criteria: using the same ART regimen as cases; current age (±5 years); and CD4 count (±50 cells). We accepted baseline CD4 counts up to six months old as long as the participant had not used hormonal contraception for the wash-out periods (as specified above) before the blood specimen was collected. Furthermore, the baseline CD4 count was only usable if the participant had been on ART for at least six months prior to that specimen being collected and if it was at least 200 cells. In addition to the criteria outlined above, we applied the following exclusion criteria for controls to better avoid possible contamination: any use of hormonal contraception between baseline CD4 and enrolment date; plans to use hormonal contraception in the next 12 months.

Regular hospital services included periodic check-ups, ART provision and periodic blood draws to measure CD4 counts. We did not interfere with clinical management of patients but did advocate for blood draws and lab tests to maintain the routine standard of care at that facility (every six months, if stable).

We aimed to recruit 60 implant users and an equal number of matched controls, based on variance of change in CD4 counts from previous research in Uganda and Zimbabwe [10] and the following assumptions: the mean change from baseline CD4 will not differ by more than 100 cells per µL compared with the non-hormonal group, two-sided 0.05 level test, 80% power to detect a difference of 105 cells per µL in the change from baseline and 15% lost to follow-up or death.

We followed the participants prospectively and recorded CD4 counts as they became available through the regular services (approximately every six months). In addition, each time participants returned for regular services, they were asked to visit the study nurse and provide an update on contraceptive use, illnesses and pregnancy. At that time, the study nurse also reviewed medical records, if feasible. At the close of the study, final interviews and medical record reviews were conducted. The following key information was transcribed to study forms: ART medications/dates, CD4 counts/dates, opportunistic infections/dates and pregnancies/dates.

The primary outcome was change in CD4 count over time, comparing implant users to their non-hormonal controls. Secondary outcomes included the incidence of pregnancy and opportunistic infections. We used generalized growth curve models and Wald chi-square tests to compare changes in CD4 counts across the study groups, which controls for time in study. Prospective CD4 measures were censored (excluded), if any of the following events occurred: change in ART, implant removal and use of hormonal contraception among controls. Analyses were conducted using SAS 9.2 (SAS Institute, Cary, NC, USA).

## Results

We successfully recruited 60 implant users, but six were later deemed ineligible for the following reasons: baseline CD4 count below 200 cells (two), missing baseline CD4 count (one) and incorrect ART (three were using tenofovir disoproxil fumarate (TDF) regimens). Recruitment of matched controls was more difficult. A total of 36 women were recruited, but three participants were later deemed ineligible for the following reasons: incorrect ART regimen, use of hormonal contraception in wash-out period and matching error. Of the 33 eligible, non-hormonal controls, 15 were matched to an additional implant user. Thus, we analyzed data on 33 non-hormonal controls and 48 implant users for a total study size of 81 women.

Because of matching, implant users and non-hormonal controls were similar in terms of age and baseline CD4 counts (Table 1). In addition, the groups were similar in terms of education, marital status and number of years on ART. Implant users had higher parity levels than non-hormonal controls. During follow-up, 44% of implant users experienced censoring events (implant removal or change in ART regimen) that made subsequent CD4 measures unusable. In contrast, 18% of non-hormonal controls had censoring events, and so the distribution of number of usable CD4 measures in the two groups differed. Nearly three-quarters of non-hormonal controls had a usable 12-month measure compared to about half of implant users.

**Table 1:** Characteristics of matched levonorgestrel implant users and non-hormonal controls

Characteristics	Implant user *N*=48	Non-hormonal control* *N*=33
Mean age (s.d.)	32.0 (5.3)	33.6 (5.4)
Mean baseline CD4 (s.d.)	420 (121)	417 (140)
Education (%)		
Less than primary	16.7	15.2
Completed primary	33.3	30.3
Completed secondary or higher	50.0	54.5
Marital status (%)		
Single	41.7	36.4
Married	58.3	63.6
Number of children (%)		
0–1	33.3	42.4
2	37.5	48.5
3+	29.2	9.1
Years on ART (s.d.)	2.84 (0.99)	2.97 (1.04)
Censoring events within 12 months (%)		
Implant removal	12.5	–
Change in ART	31.3	15.2
Adoption of hormonal contraception	–	3.0
Number of usable** CD4 measures (%)		
baseline only	22.9	0.0
1	16.7	15.2
2	47.9	60.6
3	6.3	15.2
4	6.3	9.1
Usable** CD4 counts (%), 12 months or later	47.1	72.7

* 32 were using condoms and 1 participant was not using contraception.

** Excludes CD4 measures taken after switching ART, implant removal or use of hormonal contraception among controls.

**Figure 1:**
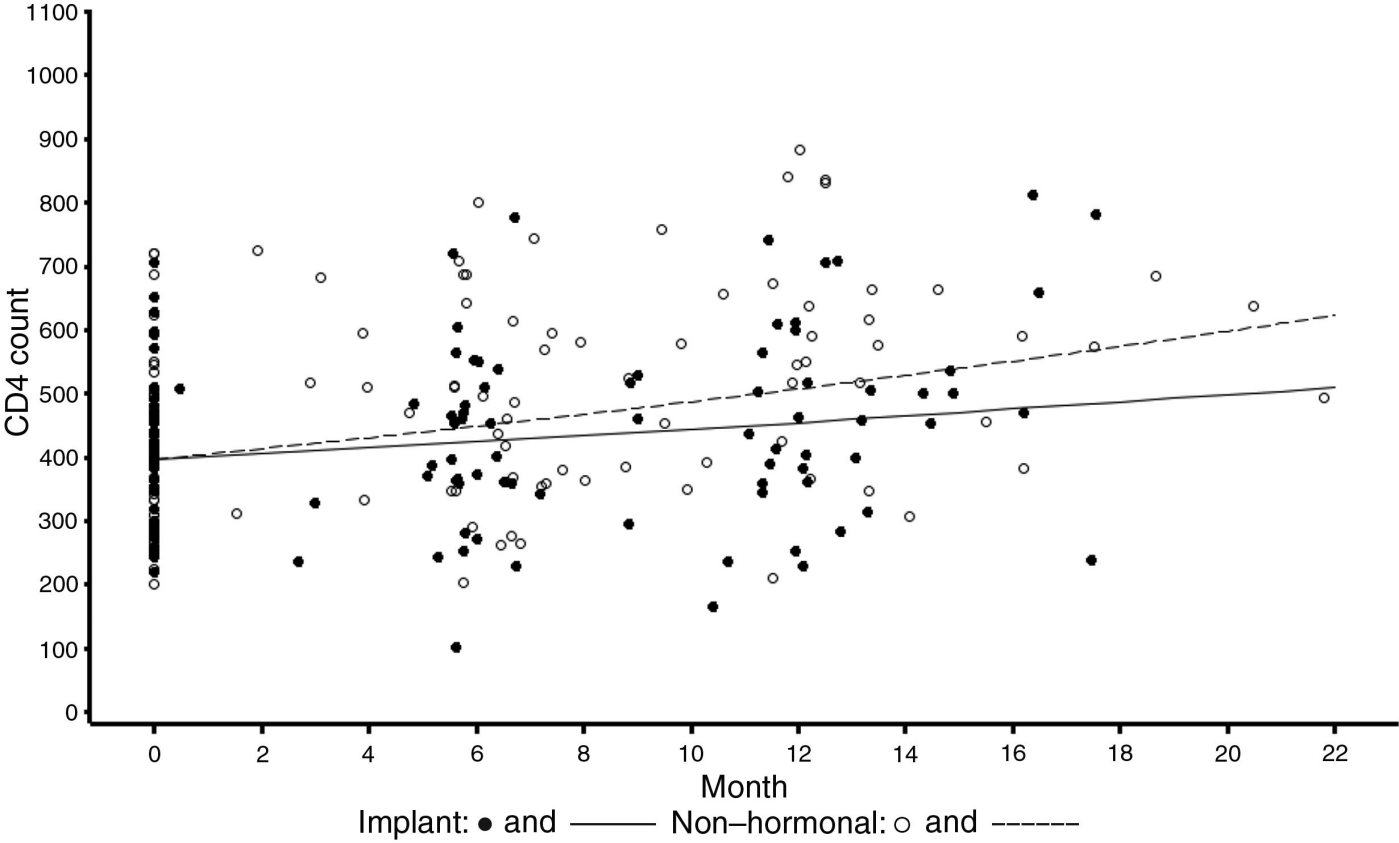
Estimated trend of CD4 count by group (*p*-value=0.44).

Over time, the trend in CD4 counts was similar for implant users and non-hormonal controls (Figure 1), with slight increases in CD4 counts in both groups. On average, there was an 86 cell per µL difference between the two groups (*p*-value=0.44), after controlling for participant duration in the study. The mean increase in CD4 was 59 for implant users and 101 for non-hormonal controls at 12 months (*p*-value=0.10). No participants died during follow-up. Six participants in the complete cohort (*n=*96) were diagnosed with opportunistic infection (two implant users and four non-hormonal control participants). The two implant users acquired oral candidiasis and herpes zoster, while infections among the non-hormonal controls included pneumonia (three) and cryptococcal meningitis (data not shown). None of the implant users and one of the non-hormonal controls became pregnant during follow-up. Of the 60 implant users enrolled, six participants (10%) had the product removed within 12 months of insertion.

## Discussion

We found that use of a levonorgestrel sub-dermal implant did not adversely affect patients on ART. CD4 counts rose slowly and equally in women using implants and in non-hormonal users. Few opportunistic infections occurred in both groups. ART did not compromise contraceptive efficacy of the implant. To our knowledge, this is the first attempt to understand how simultaneous use of a levonorgestrel sub-dermal contraceptive implant and ART may affect both HIV progression and contraceptive effectiveness.

Many studies have examined the safety of hormonal contraception among women living with HIV. Nearly all showed that use of oral contraceptives or injectable DMPA does not affect HIV progression [6]. A recent pharmacokinetic study showed ART does not affect DMPA [11]. In terms of slow-release contraceptive products, a small study by Heikinheimo *et al*., found that the levonorgestrel intrauterine device did not adversely affect women living with HIV or increase genital shedding of HIV [12]. Recently, case reports of contraceptive failure of an etonogestrel implant (while using efavirenz) have raised concerns over simultaneous use [13,14].

Our study has weaknesses, primarily related to the small study size. Though we were hoping to have about twice the number of participants, the recruitment and matching requirements proved to be more difficult than anticipated. Changing ART regimens also impacted our work and we had to censor CD4 values that were affected. Regimen switching was more common in the implant group (compared to the non-hormonal group) due to timing of follow-up visits, availability of medications and other administrative reasons, not medical reasons. For control participants, we relied on self-reports of “no hormonal use”; unfortunately this is a study weakness that has few solutions.

Our work is noteworthy for four reasons. First, we studied a contraceptive product that releases progestin automatically and with certainty; thus, we avoided reliance on self-reported data (e.g. from oral contraceptive use if that product were used in the study). Second, we matched women prospectively on key factors, including ART regimen, baseline CD4 count and age. Without this design feature, we would have risked collecting data on extremely disparate cohorts and simultaneously introducing bias. Third, we carefully recorded changes in ART and contraception to verify exposures, correctly censor data and preserve validity. Finally, our report is timely; for example, WHO recently reviewed existing evidence and concluded more research is needed on contraception and HIV [15].

## Conclusions

Sub-dermal implants are in the top tier of contraceptive effectiveness [16] and their use is increasing in sub-Saharan Africa [17]. In addition, millions of women in sub-Saharan Africa are taking ART and access to medications is increasing [18]. The results from this study provide some reassurance that levonorgestrel sub-dermal implants are safe and effective contraceptive options for women living with HIV using ART. In the broader context, increasing access to all family planning methods will help prevent unintended pregnancy and thus reduce HIV incidence from mother-to-child transmission [19]. Increasing access to sub-dermal implants through expansion of both product procurement and provider training can have tremendous public health impact.
